# DNA Methylation Levels in Mononuclear Leukocytes from the Mother and Her Child Are Associated with IgE Sensitization to Allergens in Early Life

**DOI:** 10.3390/ijms22020801

**Published:** 2021-01-14

**Authors:** Nathalie Acevedo, Giovanni Scala, Simon Kebede Merid, Paolo Frumento, Sören Bruhn, Anna Andersson, Christoph Ogris, Matteo Bottai, Göran Pershagen, Gerard H. Koppelman, Erik Melén, Erik Sonnhammer, Johan Alm, Cilla Söderhäll, Juha Kere, Dario Greco, Annika Scheynius

**Affiliations:** 1 Department of Clinical Science and Education, Karolinska Institutet, and Sachs’ Children and Youth Hospital, Södersjukhuset, SE-118 83 Stockholm, Sweden; nacevedoc@unicartagena.edu.co (N.A.); simon.merid@ki.se (S.K.M.); erik.melen@ki.se (E.M.); johan.alm@ki.se (J.A.); 2 Institute for Immunological Research, University of Cartagena, 130014 Cartagena, Colombia; 3 Department of Biology, University of Naples Federico II, 80138 Napoli, Italy; giovanni.scala@unina.it; 4 Faculty of Medicine and Health Technology, Tampere University, 33520 Tampere, Finland; dario.greco@tuni.fi; 5 Institute of Biosciences and Medical Technologies (BioMediTech), Tampere University, 33520 Tampere, Finland; 6 Department of Political Sciences, University of Pisa, 56126 Pisa, Italy; paolo.frumento@unipi.it; 7 Department of Medicine Solna, Translational Immunology Unit, Karolinska Institutet, SE-171 77 Stockholm, Sweden; soerenbruhn@hotmail.com (S.B.); anna@nixotech.se (A.A.); 8 Stockholm Bioinformatics Center, Department of Biochemistry and Biophysics, Stockholm University, Science for Life Laboratory, SE-17121 Solna, Sweden; christoph.ogris@helmholtz-muenchen.de (C.O.); erik.sonnhammer@scilifelab.se (E.S.); 9 Institute of Computational Biology, Helmholtz Center Munich, 85764 Neuherberg, Germany; 10 Institute of Environmental Medicine, Karolinska Institutet, SE-171 77 Stockholm, Sweden; matteo.bottai@ki.se (M.B.); goran.pershagen@ki.se (G.P.); 11 Section of Pediatric Pulmonology and Pediatric Allergology, Beatrix Children’s Hospital, University Medical Center Groningen, University of Groningen, 9713 GZ Groningen, The Netherlands; g.h.koppelman@umcg.nl; 12 Groningen Research Institute of Asthma and COPD (GRIAC), University Medical Center Groningen, University of Groningen, 9700 RB Groningen, The Netherlands; 13 Department of Biosciences and Nutrition, Karolinska Institutet, SE-171 77 Stockholm, Sweden; cilla.soderhall@ki.se (C.S.); juha.kere@ki.se (J.K.); 14 Department of Women’s and Children’s Health, Karolinska Institutet, SE-171 77 Stockholm, Sweden; 15 Folkhälsan Research Institute, Stem Cells and Metabolism Research Program, University of Helsinki, 00014 Helsinki, Finland; 16 Institute of Biotechnology, University of Helsinki, FI-00014 Helsinki, Finland; 17 Science for Life Laboratory, Karolinska Institutet, SE-171 65 Solna, Sweden

**Keywords:** ALLADIN, allergens, atopy, BAMSE, DNA methylation, IgE sensitization, epigenetics, maternal effects

## Abstract

DNA methylation changes may predispose becoming IgE-sensitized to allergens. We analyzed whether DNA methylation in peripheral blood mononuclear cells (PBMC) is associated with IgE sensitization at 5 years of age (5Y). DNA methylation was measured in 288 PBMC samples from 74 mother/child pairs from the birth cohort ALADDIN (Assessment of Lifestyle and Allergic Disease During INfancy) using the HumanMethylation450BeadChip (Illumina). PBMCs were obtained from the mothers during pregnancy and from their children in cord blood, at 2 years and 5Y. DNA methylation levels at each time point were compared between children with and without IgE sensitization to allergens at 5Y. For replication, CpG sites associated with IgE sensitization in ALADDIN were evaluated in whole blood DNA of 256 children, 4 years old, from the BAMSE (Swedish abbreviation for Children, Allergy, Milieu, Stockholm, Epidemiology) cohort. We found 34 differentially methylated regions (DMRs) associated with IgE sensitization to airborne allergens and 38 DMRs associated with sensitization to food allergens in children at 5Y (Sidak *p* ≤ 0.05). Genes associated with airborne sensitization were enriched in the pathway of endocytosis, while genes associated with food sensitization were enriched in focal adhesion, the bacterial invasion of epithelial cells, and leukocyte migration. Furthermore, 25 DMRs in maternal PBMCs were associated with IgE sensitization to airborne allergens in their children at 5Y, which were functionally annotated to the mTOR (mammalian Target of Rapamycin) signaling pathway. This study supports that DNA methylation is associated with IgE sensitization early in life and revealed new candidate genes for atopy. Moreover, our study provides evidence that maternal DNA methylation levels are associated with IgE sensitization in the child supporting early in utero effects on atopy predisposition.

## 1. Introduction

During the last decades, the prevalence of allergic sensitization and allergic diseases has increased worldwide. It has been hypothesized that environmental conditions modify the epigenome of immune cells, and together with present lifestyle conditions, the immune response is skewed to pro-allergic profiles [1,2]. Among the epigenetic mechanisms, DNA methylation is of interest because it is very dynamic in several immune genes early in life [3]. IgE sensitization to food and airborne allergens usually starts during early childhood and is for many children the first step in the progression to clinical manifestations such as atopic eczema, food allergy, seasonal or perennial rhinitis, and/or asthma [4]. In recent years, some studies have evaluated the association between DNA methylation and the development of allergen-specific IgE sensitization [5,6,7,8,9]. However, the cross-sectional design of these studies imposes the limitation that it is not possible to know if the observed DNA methylation differences are a consequence of the ongoing atopic reactions or precede the inception of immunoglobulin E (IgE) sensitization. This can only be addressed by profiling repeated samples from the same children from birth through childhood, before and after allergen-specific IgE antibodies are detectable. Furthermore, a relationship between maternal DNA methylation and atopic predisposition in the offspring has been observed in animal models [10,11]. However, no study has yet shown the association of DNA methylation levels in maternal cells with the presence of IgE sensitization to allergens in their children. 

The ALADDIN (Assessment of Lifestyle and Allergic Disease During INfancy) study is a prospective birth cohort [12] that aims to elucidate the role of lifestyle-related factors associated with the protection of allergy [13,14]. We used this birth cohort to conduct a genome-wide profiling of DNA methylation levels in mothers during the last trimester of pregnancy and repeated peripheral blood mononuclear cells (PBMC) samples from their children at birth at 2 years (2Y) and at 5 years (5Y) of age. The aims were (i) to study the relationship between DNA methylation levels in children and the development of IgE sensitization to allergens, and (ii) to assess if maternal DNA methylation levels are associated with IgE sensitization in their children. This study showed significant differences in DNA methylation levels between IgE-sensitized children at 5Y compared to non-sensitized children, which can be already detected in maternal PBMCs, cord blood, and at 2Y. In addition, it reveals new candidate genes predisposing to allergen sensitization involved in endocytosis, MAP kinase pathways, and the mTOR signaling pathway.

## 2. Results

**Figure 1:**
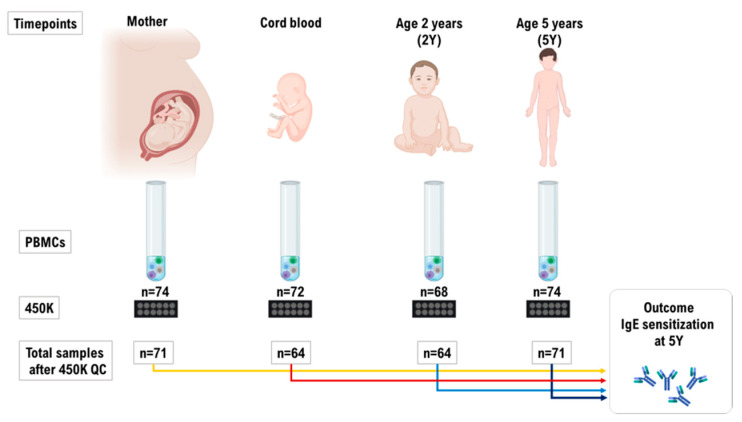
A schematic representation of the study time points, samples, and outcome. Detailed information on quality control and the excluded samples is presented in Figure S1.

We analyzed DNA methylation levels in 288 samples from 74 mother/child pairs from the ALADDIN cohort using the HumanMethylation450 BeadChip (Illumina Inc., San Diego, CA, USA) and 18 samples were removed after quality control (Figure S1). The final dataset considered for DNA methylation analysis included a total of 270 PBMC samples from the mothers obtained during pregnancy (n = 71), from cord blood (CB, n = 64), and the child at 2Y (n = 64), and 5Y (n = 71) (Figure 1). IgE sensitization to airborne allergens at 5Y of age was defined by a positive result (IgE ≥ 0.35 kU_A_/L) in the Phadiatop test (Thermo Fisher Scientific) as the study outcome (Figure 1).

The cell proportions in PBMCs were estimated in each sample by flow cytometry using the 7-color immunophenotyping kit (Table S1). They did not differ between sensitized and non-sensitized mothers or children (Table S2), or according to lifestyle (Table S3). However, since cell proportions change with age, the analysis of DNA methylation levels were performed per each time point (Figure 1). A total of 38 children were IgE sensitized (IgE ≥ 0.35 kU_A_/L) to airborne allergens at 5Y, and 17 of those were IgE sensitized to airborne and food allergens. To increase efficiency, the sensitized children were matched to non-sensitized controls as much as the dataset allowed (Table 1). Since the ALADDIN study included families with different lifestyles, this was included as a covariate in the DNA methylation analysis.

### 2.1. DNA Methylation Differences in ALADDIN

**Figure 2:**
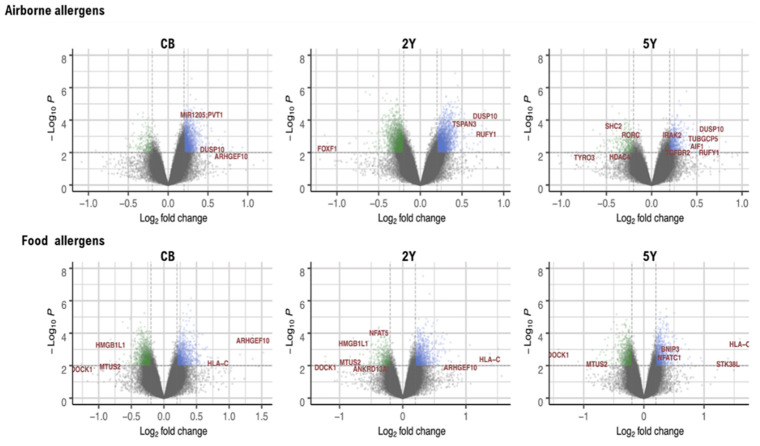
Volcano plots on the differential methylation at different time points for children sensitized to airborne and food allergens. Genes with the largest methylation differences (fold change) are presented in brown labels. Green dots represent DMPs with reduced methylation in sensitized children and blue are DMPs with increased methylation in sensitized children. Dotted lines represent the fold change threshold (0.2, vertical line) and the *p*-value threshold of <0.01 (horizontal line). CB = cord blood, 2Y = age 2 years, 5Y = age 5 years.

DNA methylation data were analyzed for differentially methylated CpG probes (DMPs) at each sampling time point (CB, 2Y and 5Y) to test their association with airborne and food allergen sensitization at 5Y. Albeit some DMPs showed a *p* value below the suggestive genome-wide significance threshold (*p* ≤ 1 × 10^−5^) or showed reproducible fold change differences between sensitized and non-sensitized children at different time points (Figure 2), they were not significant after Benjamini–Hochberg correction. Details on the DMPs associated with IgE sensitization at each sampling time point (CB, 2Y, and 5Y) with a nominal *p* < 0.01 and fold variation > 0.2 are presented in the Spreadsheet File S1. This analysis suggested that children sensitized to airborne allergens had increased DNA methylation in genes encoding the dual specificity phosphatase 10 (*DUSP10*) and the RUN and FYVE domain containing 1 (*RUFY1*), which could be detected at different time points. Moreover, it suggested that food-sensitized children had reduced DNA methylation in their CB, 2Y, and 5Y samples in the genes encoding dedicator of cytokinesis 1 (*DOCK1*) and microtubule-associated scaffold protein 2 (*MTUS2*) (Figure 2).

**Figure 3:**
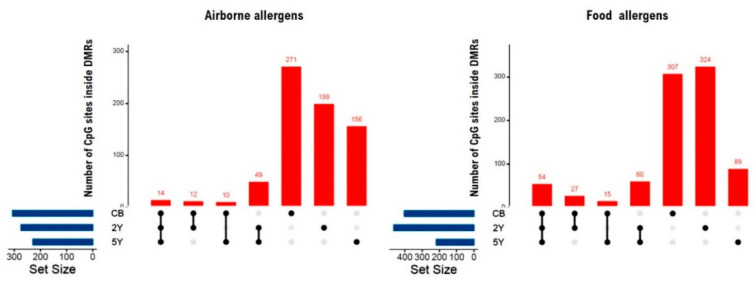
Bar plot showing the total number of CpG sites located within the significant differentially methylated regions (DMRs) associated to airborne and food sensitization in ALADDIN (Assessment of Lifestyle and Allergic Disease During INfancy). The total number of CpG sites that are associated to each phenotype at each time point is reported above the red bars. Black dots represent the intersected sets assigned to the red bar. The blue bars indicate the total number of CpGs within DMRs per time point; the overlap of cg numbers was used as proxy of DMR composition per time point. Detailed information on the CpG sites within the DMRs associated with IgE sensitization is presented in Spreadsheet File S2 (airborne allergens) and Spreadsheet File S3 (food allergens).

Then, we analyzed the association of differentially methylated regions (DMRs) with the presence of IgE sensitization in the children at 5Y, and we found significant associations that survived correction for multiple testing (Sidak *p* value ≤ 0.05). The number of CpG sites located inside the significant DMRs at each time point and their overlap is presented in Figure 3. There was a scarce overlap among the DMRs associated with IgE sensitization at different time points; for instance, only 49 CpG sites inside the regions associated with airborne sensitization and 60 CpG sites inside the regions associated with food sensitization were also observed in DMRs from samples taken at 2Y and 5Y. In addition, there were 14 CpG sites inside the regions associated with airborne sensitization and 54 CpG sites inside the regions associated with food sensitization that were found in common in CB, 2Y, and 5Y samples (Figure 3).

#### 2.1.1. DMRs Associated with IgE Sensitization to Airborne Allergens at 5 Years

We found 34 DMRs associated with IgE sensitization to airborne allergens at 5Y (Spreadsheet File S4). The most significant region was located on the *RUFY1* gene, showing increased DNA methylation in IgE-sensitized children in nine CpGs distributed in 777 base pairs (bp) (Table 2). DMRs in the genes encoding tubulin gamma complex associated protein 5 (*TUBGCP5*) and the allograft inflammatory factor 1 (*AIF1*) were also more methylated in IgE-sensitized children. The DMRs with the most significant decreases in DNA methylation in sensitized children were mapped to the dual specificity phosphatase 22 (*DUSP22*) and the *CD300A* genes (Table 2).

Then, we analyzed DNA methylation levels in PBMCs obtained from the mothers during the last trimester of pregnancy. We found 25 DMRs in maternal PBMCs associated with the presence of IgE sensitization to airborne allergens in their children at age 5Y (Spreadsheet File S5). The genes annotated to these DMRs included arachidonate 12-lipoxygenase pseudogene 2 (*ALOX12P2*), cysteine-rich protein 2 (*CRIP*), and S100 calcium binding protein A13 (*S100A13*), among others. We also observed significant association with a DMR mapped to the *RUFY1* gene in maternal PBMCs. A summary of the top 10 DMRs in maternal PBMCs is presented in Table 3. 

#### 2.1.2. DMRs Associated with IgE Sensitization to Food Allergens at 5 Years

Seventeen children had in addition to airborne sensitization concomitant IgE sensitization to food allergens (Table 1). DNA methylation analysis of PBMCs from children at 5Y revealed 38 DMRs associated with IgE sensitization to food allergens at 5 years (Spreadsheet File S4). Only five DMRs were also associated with airborne allergen sensitization. These DMRs mapped to *CD300A* and the PRR34 antisense RNA 1 (*PRR34-AS1*); or, they were located in the proximity of the genes: MEF2-activating motif and SAP domain containing transcriptional regulator (*MAMSTR*), dedicator of cytokinesis 1 (*DOCK1*), and BR serine/threonine kinase 2 (*BRSK2)*. The other 33 DMRs were only found significant in children with food sensitization (Spreadsheet File S4). A summary of the top DMRs associated with food sensitization is presented in Table 4. Albeit the top significant DMRs showed increased methylation in sensitized children, the 10 DMRs with reduced DNA methylation showed more remarkable differences between food sensitized and non-sensitized children (Table 4).

#### 2.1.3. Biological Pathways Related to Differentially Methylated Regions and IgE Sensitization

**Figure 4:**
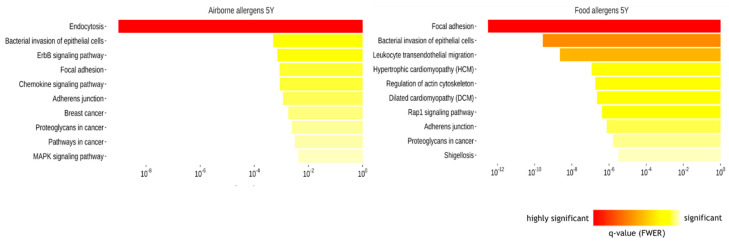
The relationship between DNA methylation changes with biological pathways. The top 10 enriched pathways for the genes within DMRs associated with airborne and food allergen sensitization at age 5Y in ALADDIN.

To elucidate the biological pathways related with the DNA methylation differences, we derived the genomic coordinates of the DMRs associated with airborne and food allergen sensitization at 5Y and annotated the most proximal gene within 500 bp. The resulting gene lists were used to search for significantly overrepresented KEGG (Kyoto Encyclopedia of Genes and Genomes v82.1) pathways [15]. The significantly enriched pathways in each comparison are shown in Figure 4. The most significant pathway associated with airborne allergen sensitization was endocytosis (q = 9.5 × 10^−10^). Regarding the genes related with this pathway, 20 were connected to *RUFY1*, 12 genes were connected to the tyrosine kinase encoding gene *TNK2*, 7 genes were connected to ubiquitin conjugating enzyme *UBE2J2,* and 3 genes were connected to *DOCK1*. The most significant pathways related with food allergen sensitization were focal adhesion (q = 3.0 × 10^−13^), bacterial invasion of epithelial cells (q = 2.7 × 10^−10^), and leukocyte transendothelial migration (q = 2.2 × 10^−9^). The genes implicated in these pathways with the largest number of connections were septin 9 (*SEPT9)*, insulin-like growth factor 1 receptor (*IGF1R*) and *DOCK1.*

**Figure 5:**
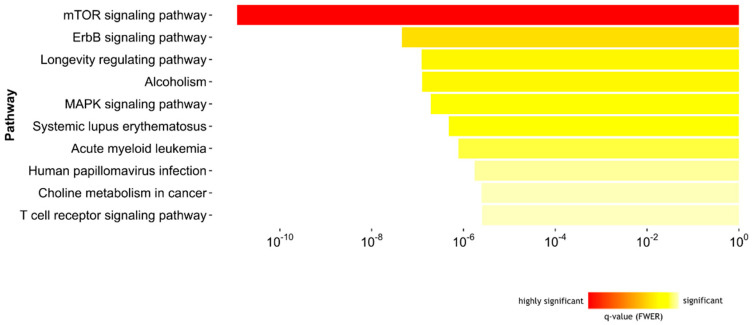
The relationship between DNA methylation changes with biological pathways. The top 10 enriched pathways for the genes within maternal DMRs associated with airborne allergen sensitization at age 5Y in ALADDIN.

Genes mapped to DMRs in maternal PBMCs associated with airborne sensitization at 5 years were enriched in pathways of mTOR signaling pathway (q = 1.3 × 10^−11^) and MAP kinase signaling pathway (q = 4.4 × 10^−6^) (Figure 5).

### 2.2. Replication in the BAMSE Cohort

Then, we aimed to replicate the differences in DNA methylation detected in ALADDIN children at 5Y in another cohort. Thereby, we retrieved the DNA methylation levels of the CpG sites located within the start and end positions of the DMRs detected in ALADDIN (based on the genomic coordinates) and analyzed their association with IgE sensitization to airborne and food allergens in whole blood samples collected at about 4Y in 256 children from the BAMSE cohort (Table S4). After quality control, we obtained data on 238 CpG sites within the 34 DMRs associated with airborne allergen sensitization and 256 CpG sites in the 38 DMRs associated with food sensitization. Of these, 13 CpG sites (5.5%) were associated with airborne allergen sensitization and 25 CpG sites (9.8%) were associated with food allergen sensitization in BAMSE children (nominal *p* < 0.05) (Spreadsheet file S6). CpG sites associated with airborne sensitization in BAMSE mapped to *AIF1* (cg04812347), *TUBGCP5* (cg06756169), and *CCR9* (cg06519172, cg10475172). Those associated with food allergen sensitization mapped to *TTL10* (cg25544075), *USP6NL* (cg10853431), *WRAP53* (cg21050342, cg02166782, cg13169780), *SEPT9* (cg03568017, cg25690715), *ZNF577* (cg22472290, cg13393830, cg23010048, cg22331349), *COMMD2* (cg01946548), and *DAAM2* (cg27190145). Although DNA methylation differences could be detected in these CpG sites in both cohorts, their nominal *p* value in BAMSE did not pass Bonferroni correction (Spreadsheet file S6). The exact DMRs as detected in ALADDIN were not replicated in the BAMSE dataset.

Then, we performed a meta-analysis for the top 1% DMPs using the discovery ALADDIN and replication BAMSE cohorts together and found 1966 CpG sites for airborne allergen sensitization and 2985 CpG sites for food allergen sensitization that had a nominal *p*-value < 0.05 and same direction of effect. Of these, 11 DMPs for airborne sensitization and 40 DMPs for food sensitization survived Bonferroni correction for multiple testing (Spreadsheet file S6). This analysis replicated the association of cg01623485 in *BRSK2* with food sensitization (METAL *p* value = 1 × 10^−5^).

## 3. Discussion

Few prospective studies have analyzed DNA methylation levels in repeated blood samples from birth to early in life in relation with IgE levels against allergens [16]; however, none has analyzed yet DNA methylation in human maternal cells during pregnancy and its association with IgE sensitization in the child. Here, we analyzed DNA methylation levels in PBMCs collected from the mother, cord blood, and their child at 2Y and 5Y, and we tested the association with IgE sensitization at 5Y as outcome (Figure 1). We found differentially methylated regions (DMRs) associated with IgE sensitization to airborne and food allergens in children at 5Y. Since we analyzed repeated DNA samples from the same child, we also detected significant DNA methylation differences in cord blood and 2Y samples that presumably preceded the IgE switching. Most remarkably, we detected DMRs in maternal cells that were associated with sensitization in their children at 5Y. Considering that there were no significant differences in cell proportions of PBMC samples according to sensitization or lifestyle (Tables S2 and S3), our results are most probably not affected by cell heterogeneity [17].

**Figure 6:**
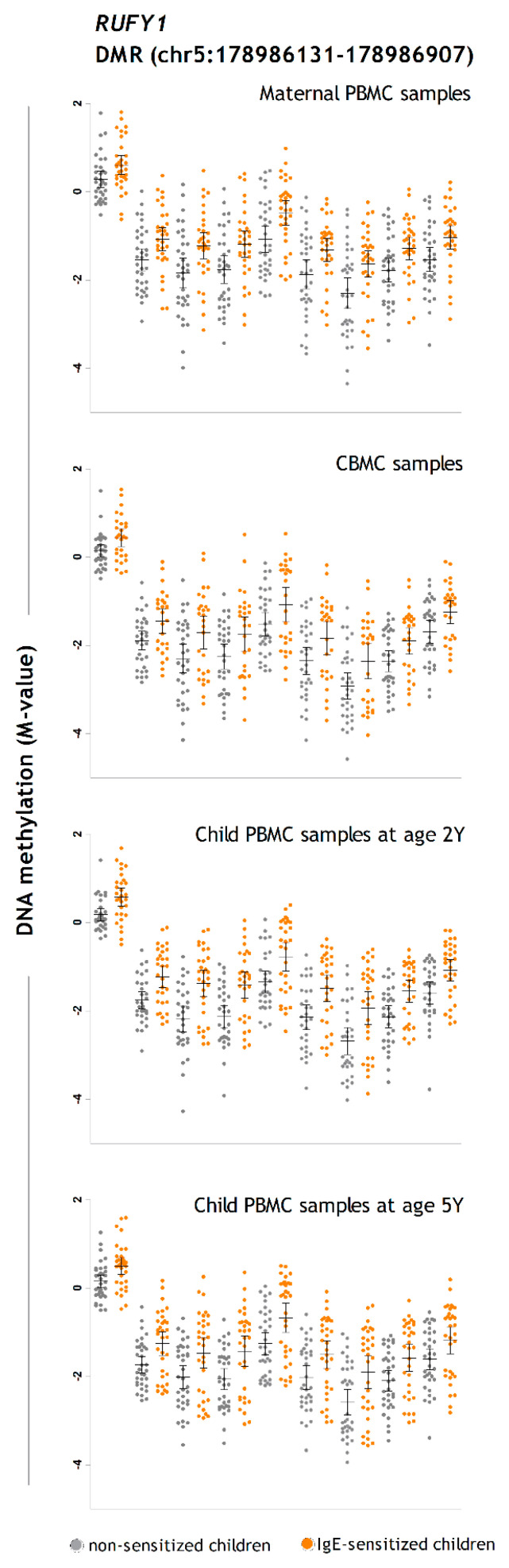
DNA methylation levels in nine CpG sites located in the DMR in *RUFY1* gene per time point. From left to right: cg19626725, cg00080972, cg21226059, cg14820908 (located in the upstream shore) and cg02136620, cg09060608, cg05457628, cg22764044, and cg26516362 (in the CpG island). Detailed information on the genomic annotation of these CpG sites is presented in Spreadsheet File S2. Error bars represent mean and 95% confidence interval. Chr: chromosome.

The discovered DMRs mapped to several immune related genes and revealed exciting new candidates for atopy predisposition (Table 5). For instance, we observed consistent differences in DNA methylation between sensitized and non-sensitized children at different time points in the top significant region mapped to *RUFY1* (Figure 6)*,* which is a gene encoding a protein that binds to phosphatidylinositol-3-phosphate and plays a role in early endosomal trafficking, tethering, and fusion through interactions with small GTPases [18]. In addition, in *DUSP22*, a member of the dual specificity phosphatases involved in controlling the outcome of innate immune responses due to context-dependent expression and the selective inhibition of mitogen-activated protein kinases (MAPK) [19] and T-cell mediated immunity [20]. Moreover, the analysis of DMRs in maternal PBMCs associated with the presence of IgE sensitization in the child at 5Y revealed a region in which its closest gene encodes the regulatory associated protein of mTOR, complex 1 (*RPTOR*) (Spreadsheet File S5). DNA methylation differences in this gene were initially observed by Martino et al. in purified naïve CD4^+^ T cells from children with food allergy [8] and are now replicated in PBMC samples in this study. The connection of *RPTOR* with IgE sensitization needs to be explored in detail, since it is implicated in antibody secretion [21], dendritic cell homeostasis [22], tuft cell differentiation, and initiation of type 2 immunity [23], as well as in bronchial hyperreactivity in asthmatic patients [24].

Common gene pathways in the analysis of maternal and children DMRs included ErbB signaling and MAPK signaling, suggesting that epigenetic disturbances in genes implicated in these processes may contribute to atopy predisposition through different time points.

According to the natural history of allergic diseases and sensitization profiles derived from several European cohorts, it has been established that airborne allergens are the most frequent sensitizers in children around age 5Y [17]; thereby, we used the presence or absence of this sensitization as the index phenotype for the selection of participating children in ALADDIN. Albeit a good marker to define the trait of interest, these inclusion criteria imply that all children regarded as cases are airborne allergen sensitized. The presence of sensitization to food allergens, data that were available for this study after the selection of cases and controls and the DNA methylation analysis, co-occurs with airborne sensitization. Therefore, our design only permits dissecting DNA methylation signatures associated with sensitization to food allergens on top of other atopy predisposing loci. The results clearly show that there are several DMRs associated with food sensitization that are biologically plausible to predispose those children to react to food allergens in contrast with children that are only sensitized to airborne allergens. This study revealed for the first time that DNA methylation levels in *CD300A* were lower in IgE-sensitized children either to airborne allergens (Table 2) or to both, airborne plus food allergen sensitization (Table 4), supporting previous studies suggesting regulatory functions of this receptor on atopy and IgE-mediated, allergic inflammation [25,26,27]. Our results may also help to understand the biology of why some individuals are naturally predisposed to become IgE polysensitized. Nevertheless, given the characteristics of the ALADDIN families, further studies are needed to validate the generalizability of our findings in other populations. 

**Figure 7:**
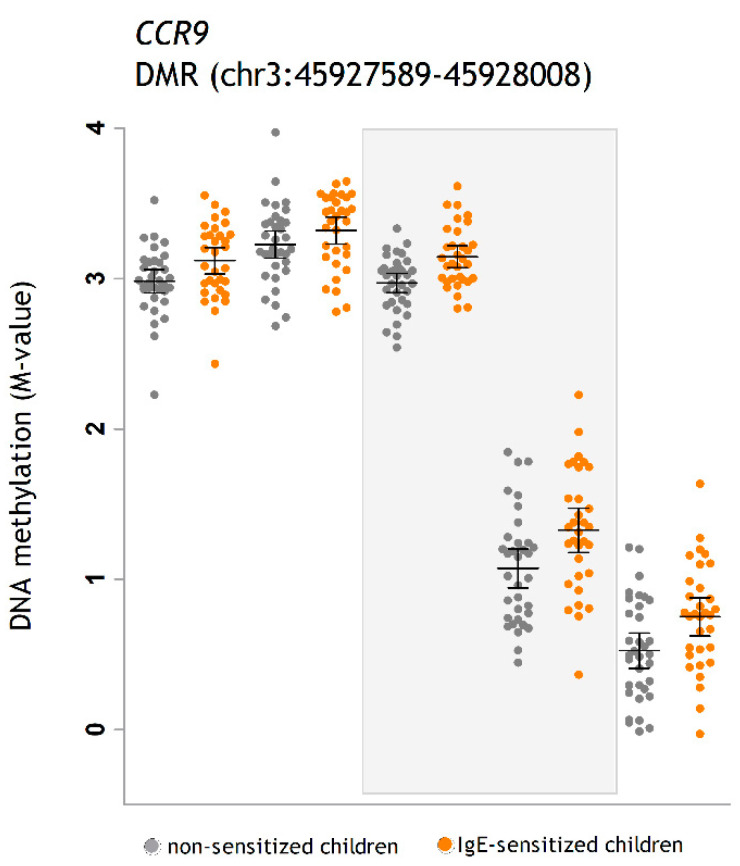
DNA methylation levels in five CpG sites located in the DMR in *CCR9* gene per time point. From left to right: cg27198997, cg09033997, cg06519172, cg10475172, and cg17642041. Detailed information on the genomic annotation of these CpG sites is presented in Spreadsheet File S2. The gray square points out the two CpG sites that were also replicated in BAMSE at nominal *p* value < 0.05 (cg06519172, cg10475172, Spreadsheet File S6). Error bars represent mean and 95% confidence interval. Chr: chromosome.

Here, we also detected significant associations in the replication BAMSE cohort for some CpG sites within the DMRs found in ALADDIN. Some of the CpG sites inside DMRs associated with airborne allergen sensitization in ALADDIN were also associated with airborne allergen sensitization in BAMSE and mapped to genes such as *TUBGCP5*, *AIF1*, claudin 14 (*CLDN14*), and *CCR9* (Table 2 and Spreadsheet S6). In addition, some CpG sites inside DMRs for food sensitization in ALADDIN were also associated with food sensitization in BAMSE and mapped to genes such as *TTL10*, *COMMD2*, *SEPT9*, *DAAM2*, *ZNF577*, and *USP6NL* (Table 4 and Spreadsheet file S6), suggesting that these loci may be implicated in type 2 immunity and IgE sensitization. While these results add robustness to our observations, design differences between ALADDIN and BAMSE cohorts should not be neglected, as they might explain why only a fraction of our results could be retrieved in common between the datasets. These include a different age for sample collection (4 years in BAMSE vs. 5 years in ALADDIN), different sample composition (whole blood in BAMSE vs. PBMC in ALADDIN), and differences in recruitment areas and family lifestyles. Still, one of the genes showing differences in DNA methylation in both datasets was *CCR9* (Figure 7). This is a G protein-coupled receptor expressed on several immune cells, including dendritic cells, CD4^+^ T cells, and B cells. CCR9 in dendritic cells drives the differentiation of Foxp3^+^ Tregs and suppresses the allergic IgE response in the gut [28]; and it also has been found as a key regulator of the early phases of airway allergic inflammation [29], with CCR9^+^ lymphocytes enhance airways allergic inflammation in mice [30]

This study has several strengths and limitations. ALADDIN is in many aspects a unique prospective birth cohort with the recruitment of families already during pregnancy with longitudinally collected blood samples and objective measurements of allergen sensitization not only in the children but also in both parents and with well-characterized lifestyles [12]. Repeated longitudinally blood sampling in the children is a strength, but at the same time, the requirement of sample availability at all time points limited the number of families that could be included in this study. The design also limited the number of children, since we decided to include equal numbers of sensitized and non-sensitized children matched as much as the dataset allowed (Table 1). The fold differences between groups were small, but this is inherent to the type of signals being investigated, since DNA methylation differences in complex diseases are usually in the range of 5 to 15%. 

A limitation is that since we analyzed PBMC samples, we cannot attribute these changes to a particular mononuclear cell type. Previous studies by D. Martino et al. [8,31] and others [32,33] revealed that PBMCs and CD4^+^ T cells might be the most relevant cells contributing epigenetic disturbances in allergy. Moreover, albeit eosinophils are truly relevant for allergy physiopathology, our previous studies revealed that blood circulating myeloid cells are largely unmethylated and under constant renewal [17], while T lymphocytes exhibit complex methylation patterns that may be more relevant for epigenetic modifications associated with immune memory and IgE sensitization [34]. Further studies are needed to validate these associations and evaluate their mechanistic implications in type 2 immunity and IgE synthesis. 

In conclusion, we found significant differences in DNA methylation levels between children IgE sensitized to airborne and food allergens at 5Y of age compared to non-sensitized children. Our analysis revealed new candidate genes predisposing to allergen sensitization including *RUFY1* and those related with the mTOR signaling pathway and the MAP kinase pathways. The differences in DNA methylation associated with IgE sensitization in the children at 5 years of age can be detected already in maternal PBMCs, cord blood, and at age 2Y. Our results also suggest that predisposition to synthesize IgE towards food allergens imply an additional group of genes that may influence how a child senses, recognizes, and processes these types of allergens. Further studies are needed to characterize the role of the differentially methylated regions in the development of IgE sensitization to allergens and the functional effects of these DNA methylation differences. The catalog of atopy predisposing genes revealed by this study opens new avenues for diagnosis and therapy and constitutes a resource of putative biomarkers of atopy in future studies.

## 4. Materials and Methods 

### 4.1. Study Population and Family Selection

Seventy-four families were selected from the birth cohort ALADDIN of 330 children from families recruited in the region of Stockholm, Sweden between 2004 and 2007 [12]. To obtain a matched case-control study, we first selected all children within the ALADDIN study with a positive IgE value (IgE ≥ 0.35 kU_A_/L) to a mix of aeroallergens by the age of 5Y (Phadiatop™, Thermo Fisher Scientific, Uppsala, Sweden). Then, we evaluated the availability of PBMC samples from their mothers at the third trimester of pregnancy and repeated PBMC samples from their children at birth (cord blood (CB)) and at 2Y and 5Y). Based on these criteria, 38 families from the three lifestyle groups in the ALADDIN cohort—anthroposophic, partly anthroposophic, or non-anthroposophic—were available for DNA methylation profiling (Table 1). Non-IgE sensitized children were selected as controls based on a negative Phadiatop™ (IgE below 0.35 kU_A_/L) at 5Y of age and no antecedents of asthma, allergy, or eczema and the availability of PBMC samples. To enhance efficiency [35], the controls were selected to match maternal age at delivery, parental IgE sensitization (Phadiatop), and parental smoking during pregnancy, lifestyle group, and the child’s gender as much as the dataset allowed, resulting in 36 control families (Table 1). Data on sensitization to food allergens at 5Y as measured by IgE-serology (fx5, Thermo Fisher Scientific) were added later when the DNA methylation results were analyzed. Demographic data were compared by Chi-square test and *t*-test using IBM SPSS Statistics for Windows, 22.0 (Armonk, NY, USA). The study was conducted in accordance with the Declaration of Helsinki and was approved by the Regional Ethical Review Board in Stockholm (project Dnr 474/01, 2002-01-07, and Dnr 182, 2010/1811-32). All parents gave their written informed consent for inclusion.

### 4.2. Preparation of Peripheral Blood Mononuclear Cells (PBMC)

Collected heparinized blood was diluted, layered over Ficoll-Paque-PlusTM (GE Healthcare Biosciences AB, Uppsala, Sweden), and centrifuged for 30 min at 400× *g* in a swing-out rotor. PBMCs were collected from the sample-medium interface, washed twice with PBS, and re-suspended in freezing medium consisting of RPMI 1640 (HyClone, Logan, UT, USA) supplemented with 10% dimethyl sulfoxide (DMSO, Merk, Darmstadt. Germany) and 10% bovine growth serum (BGS, HyClone) at a density of 10 × 10^6^ cells per mL. Cryotubes were filled with 2 mL cell suspension and placed in a freezing container filled with isopropanol, stored for 2 h at −80 °C, and then transferred to −150 °C for storage.

### 4.3. DNA Extraction and DNA Methylation Profiling

Frozen PBMCs were thawed by incubating the cryotubes in a water bath at 37 °C for 3 min and thawed with cell-culture media. To assess cell viability, 20 µL of the cell-suspension was analyzed in a Countess cell-counter (Invitrogen, Carlsbad, CA, USA). The mean viability in PBMCs from mothers and children at 2 years (2Y) and 5 years (5Y) after birth was around 90% and in CB samples 76.5% ± 1.8%. For DNA extraction, 5 × 10^6^ PBMC of viable cells as determined by trypan blue staining were pelleted per vial at 300× *g* for 10 min, frozen with dry ice, and stored at −80 °C. Genomic DNA were extracted using the QIAamp DNA Mini kit (Qiagen, Hilden, Germany). DNA concentrations were measured by Qubit 2.0 (Thermo Fisher, Darmstadt, Germany), and quality was verified by the A260/A280 ratio using nanodrop (Thermo Fisher) with an optical density cut-off of 1.8. DNA samples were diluted to 11 ng/μL, and 500 ng were bisulfite treated using the EZ-96 DNA Methylation™ kit (Zymo Research Corp., Irvine, CA, USA) according to manufacturer’s instructions in four 96-well plates. DNA samples of low and high methylation were included as controls (EpiTect Control DNA, QIAGEN, Hilden, Germany). Two technical replicates were placed on each plate to assess inter-array correlations. Denatured bisulfite-treated DNA was amplified, fragmented, and hybridized onto the HumanMethylation450 BeadChip (Illumina Inc., San Diego, CA, USA) following the manufacturer’s instructions at the Mutation Analysis Core Facility (MAF, Karolinska Institutet, Stockholm, Sweden).

### 4.4. Flow Cytometry Analysis 

Given that PBMCs are a mixture of white blood cells, cell proportions need to be considered when analyzing DNA methylation data [17]. We used an aliquot of 0.5 × 10^6^ of the thawed PBMCs to measure cell proportions by staining with the 7-color Immunophenotyping kit (Miltenyi Biotech, Gladbach, Germany) and analyzing them by flow cytometry (Fortessa, Becton Dickinson, NJ, USA). Detailed information on antibodies is described in Table S1. The antibody staining was done for 30 min at 4 °C in the dark. After staining, cells were washed with 2 mL FACS buffer (350× *g*, 8 min, 4 °C) re-suspended in 200 µL FACS buffer and kept on ice. For every sample, at least 5000 events were detected After gating based on CD45^+^ cells, cell proportions for T cells, B cells, monocytes, and neutrophils were expressed as a percentage of the total sample. All statistical analyses for cell count and individual variables were performed using the R-software version 3.0 (https://www.r-project.org/). The Kruskal–Wallis test was used for comparison of cell counts between the three different lifestyle groups and *t*-test (unpaired) for comparison between sensitized and non-sensitized individuals. A *p* value < 0.05 was considered as statistically significant.

### 4.5. Bioinformatics Analysis of DNA Methylation Data

Raw measurements from Illumina IDAT files were imported in R and normalized by the SWAN method [36]. Following quality control, a total of 18 samples were removed from the original dataset: 11 because of low intensity signal, 5 as their raw hyper-methylation values were strongly deviated toward low intensity ratios, and 2 for contamination with maternal DNA (Figure S1). The final dataset for DNA methylation analysis included 270 PBMC samples from the mothers obtained during pregnancy (MB), cord blood (CB), and the child at 2Y and at 5Y (Figure 1). Probes with a detection *p* value of less than 0.01 in all samples (n = 4308) were removed as well as CpGs whose interrogation or elongation was affected by SNPs. A final filtering was applied to remove CpG sites reported to be affected by cross-hybridizing probes (n = 29,233) [37]. After pre-processing, we ended up with 437,800 CpG sites for further analysis. M-values were extracted and checked for eventual batch effects among samples by means of principal component analysis. Two technical batches were found to significantly affect the data: plate and slide position. Data were adjusted for these two batches by sequentially applying the ComBat method [38]. DNA methylation levels were expressed as M-values. In order to find CpG sites associated in each life stage with sensitization to aeroallergens or food allergens at 5Y, three independent comparisons were performed: one for each age stage (CB, 2Y and 5Y), where DNA methylation in PBMC of IgE sensitized children was compared to DNA methylation of PBMC in non-sensitized children using *limma* [39]. In each analysis, the regression formula included methylation level of each CpG probe as the dependent variable, while sensitization to aeroallergens or food allergens was used as the main covariate with gender, lifestyle, parental sensitization, and mother’s smoking during pregnancy as additive adjustment covariates. Given the number of covariates in the model and since there were no significant differences in cell proportions of PBMC samples between sensitized and non-sensitized mothers or children (Table S2), or according to lifestyle (Table S3), we did not correct by cell proportions. We considered a CpG as associated with sensitization to airborne allergens or food allergens at each time point if in the corresponding comparison the nominal *p* value was lower than 0.01 and the absolute value of fold variation was greater than 0.2. These sites were named differentially methylated probes (DMPs). Then, the nominal *p* values were corrected for multiple testing using the Benjamini–Hochberg false discovery rate (FDR) method [39].

### 4.6. Differentially Methylated Regions (DMRs)

DMRs were obtained using comb-p tool. All *p*-values associated to the tested CpGs were adjusted based on the significant values of nearby CpG sites by means of SLK (Stouffer–Liptak–Kechris) correction to mitigate the effects of false positives [40]. Then, the SLK-adjusted *p* values were used to define differentially methylated regions using a sliding window of 500 bp. For each defined region, a *p* value was thereafter computed by combining the SLK adjusted *p* values of the contained CpGs into a Stouffer–Liptak *p* value. Then, correction for multiple testing on region *p* values was applied by using 1-step Sidak correction as indicated on comb-p guidelines [40]. 

### 4.7. Pathway Enrichment Analysis 

For pathway analysis, we analyzed genes harboring DMRs associated with IgE sensitization to airborne and food allergens at 5Y. To obtain gene sets, we associated the coordinates of the DMRs to genes by considering regions with a Sidak *p* value ≤ 0.05, an upstream distance of 500 bp from the closest gene TSS (promoter CpG), as well as a similar direction on the methylation levels for the contained CpGs (hypo- or hyper-methylated). The resulting gene lists were analyzed for pathway enrichment against the 304 pathways in KEGG (Kyoto Encyclopedia of Genes and Genomes v82.1) using the online web service Pathwax [41], which is based on the novel network pathway annotation tool BinoX [42]. The algorithm assesses the statistical significance of “pathway gene-set” enrichment by evaluating the number of interactions between genes within a genome-wide functional association network. Pathwax corrects the derived *p* values into FWER values for multiple testing using the Bonferroni correction. Pathways with an estimated q-value ≤ 0.05 were selected as significantly enriched.

### 4.8. Replication Analysis

The association between DNA methylation and IgE sensitization to airborne and food allergens was analyzed in whole blood samples from 256 children collected at about 4Y in the BAMSE cohort (Table S4) using robust linear regression. BAMSE is a prospective population-based cohort study of children recruited at birth and followed during childhood and adolescence. Details of the study design, inclusion criteria, enrollment, and data collection are described elsewhere [43]. At 4Y, the children were invited to a clinical examination including blood sampling. Serum IgE antibodies to inhalant allergens were analyzed with Phadiatop (a mixture of cat, dog, horse, birch, timothy, mugworth, *Dermatophagoides pteronyssinus,* and *Cladosporium* allergens), and to the most common food allergens with the IgE fx5 foodmix (a mixture of milk, egg white, soya bean, peanut, fish, and wheat allergens), in the Pharmacia CAP System^TM^ (Pharmacia Diagnostics AB, Uppsala, Sweden). A positive result was defined as ≥0.35 kU_A_/L [43]. Details on DNA extraction and DNA methylation analyses (Illumina 450k) have been presented elsewhere [44]. DNA methylation data were preprocessed using the *minfi* package [45], and normalized using the DASEN method from the watermelon package [46]. Covariates included in the adjusted models were age, child’s gender, batch effect, and cell counts using estimated cell type proportions calculated using the Houseman method [47]. Inverse variance-weighted fixed effects meta-analysis was done with METAL to compare results in ALADDIN and BAMSE [48]. A *p* value below the suggestive genome-wide significance threshold of 1.0 × 10^−5^ or *p* < 0.05 after Bonferroni correction was considered significant. 

### 4.9. Data Availability

The data that support the findings of this study are available in the Supplementary Materials of this article. Raw data that support the findings of this study are available on request to the corresponding author.

## Figures and Tables

**Table 1:** Demographic data of parent–child pairs included in this study.

Variable	Non-Sensitized to Airborne Allergens at Age 5 Years (n = 36)	IgE Sensitized to Airborne Allergens ^1^ at Age 5 Years (n = 38)	*p* Value ^3^
Child male gender, n (%)	20 (55.6)	20 (52.6)	0.80
IgE sensitized to food allergens ^2^ at 5Y, n (%)	0 (0)	17 (44.7)	<0.0001
Lifestyle, n (%)			
Anthroposophic	9 (25)	9 (23.7)	0.95
Partly anthroposophic	13 (36.1)	15 (39.5)	
Non-anthroposophic	14 (38.9)	14 (36.8)	
Maternal age in years, mean (s.d.)	31.4 ± 4.2	31.1 ± 5.5	0.82
Mother sensitized to airborne allergens, n (%)	9 (25)	12 (31.6)	0.53
Father sensitized to airborne allergens, n (%)	17 (48.6)	21 (58.3)	0.41
Mother smoked during pregnancy, n (%)	0	5 (13.5)	0.054
Father smoked during pregnancy, n (%)	6 (16.7)	10 (27)	0.28

^1^ Sensitization to airborne allergen: IgE level ≥ 0.35 kU_A_/L for at least one of the nine aeroallergens analyzed using Phadiatop (Thermo Fisher Scientific). ^2^ Sensitization to food allergen: IgE level ≥ 0.35 kU_A_/L for at least one of the six food allergens analyzed using a food mix, fx5 (Thermo Fisher Scientific). ^3^ *p* value was calculated by Chi-square, except for maternal age, which was calculated by *t*-test; s.d.: standard deviation.

**Table 2:** Top 10 of differentially methylated regions (DMRs) in child PBMCs associated with IgE sensitization to airborne allergens at 5 years.

Chr	Start	End	Width bp	Number of CpGs	Gene Name	Location	T	*p*-Value	Sidak *p*-Value
chr5	178,986,131	178,986,907	777	9	*RUFY1*	Promoter	4.41	1.6 × 10^−10^	9.1 × 10^−8^
chr19	15,121,204	15,121,597	394	9	*CCDC105*	Promoter	1.22	6.4 × 10^−9^	0.000007
chr1	115,397,374	11,5397,617	244	5	*SYCP1*	Promoter	0.99	7.7 × 10^−9^	0.00001
chr6	291,882	292,597	716	7	*DUSP22*	Promoter	−5.31	6.3 × 10^−8^	0.00003
chr15	22,833,149	22,833,803	655	11	*TUBGCP5*	Promoter	3.42	7.5 × 10^−8^	0.00005
chr17	72,462,164	72,462,636	473	6	*CD300A*	Promoter	−1.56	2.6 × 10^−7^	0.0002
chr13	24,144,483	24,144,986	504	6	*TNFRSF19*	Promoter	1.31	3.4 × 10^−7^	0.0002
chr6	31,583,458	31,584,224	767	5	*AIF1*	Promoter	1.97	8.0 × 10^−7^	0.0004
chr19	49,223,814	49,224,166	353	5	*RASIP1*	3′ UTR	−1.02	5.7 × 10^−7^	0.0007
chr1	2,120,985	2,121,522	538	5	*FAAP20*	3′ UTR	−1.94	9.4 × 10^−7^	0.0007

Chr = chromosome; coordinates based on the human GRCh37/hg19 assembly; bp = base pair.

**Table 3:** Top 10 of differentially methylated regions (DMRs) in maternal PBMCs associated with IgE sensitization to airborne allergens in children at 5 years.

Chr	Start	End	Width bp	Number of CpGs	Gene Name	Location	T	*p*-Value	Sidak *p*-Value
chr17	6,796,745	6,797,772	1028	9	*ALOX12P2*	Promoter	−2.54	1.1 × 10^−18^	4.7 × 10^−16^
chr14	105,944,604	105,945,700	1097	7	*CRIP2*	3′ UTR	−2.04	2.7 × 10^−11^	1.1 × 10^−8^
chr5	178,986,131	178,986,907	777	9	*RUFY1*	Promoter	4.32	1.0 × 10^−8^	0.000005
chr1	153,599,479	153,600,157	679	8	*S100A13*	Promoter	−3.21	1.0 × 10^−8^	0.000007
chr6	28,911,468	28,912,167	700	12	LINC01556 *	Non-coding Exon	−3.23	4.0 × 10^−8^	0.000025
chr19	50,249,464	50,249,928	465	6	TSKS *	intron	−1.52	4.3 × 10^−8^	0.000040
chr11	43,290,958	43,291,211	254	5	*HNRNPKP3*	Promoter	1.24	2.7 × 10^−7^	0.0004
chr22	30,901,249	30,901,648	400	4	*SEC14L4*	Promoter	−0.95	0.000003	0.0032
chr10	77,164,987	77,165,751	765	7	*ZNF503-AS2*	Intron	−1.30	0.000007	0.0040
chr20	36,148,604	36,149,272	669	30	*NNAT*	Promoter	−3.71	0.000007	0.0050

* Gene with the closest transcription start site to the DMR. Chr = chromosome; bp = base pair.

**Table 4:** Differentially methylated regions (DMRs) in children PBMCs at 5 years associated with IgE sensitization to food allergens at 5 years.

Chr	Start	End	Width bp	Number of CpGs	Gene Name	Location	T	Z *p*-Value	Z Sidak *p*-Value
**DMRs detected with increased DNA methylation levels in children sensitized to food allergens**									
chr10	135,278,717	135,279,148	432	5	*SCART1*	Promoter	1.63	1.4 × 10^−10^	1.4 × 10^−7^
chr19	37,825,307	37,825,680	374	7	*HKR1*	Promoter	2.40	1.6 × 10^−8^	0.000019
chr1	1,108,820	1,109,984	1165	8	*TTLL10*	Promoter	2.65	7.1 × 10^−8^	0.000027
chr4	74,847,646	74,847,830	185	7	*PF4*	Promoter	2.82	1.4 × 10^−8^	0.00003
chr13	88,328,009	88,328,295	287	4	*SLITRK5*	Exon	1.67	9.3 × 10^−8^	0.0001
chr13	26,586,254	26,587,012	759	7	*ATP8A2*	Exon	1.91	3.6 × 10^−7^	0.0002
chr8	70,980,488	70,981,069	582	3	*PRDM14*	Exon	0.54	2.7 × 10^−7^	0.0002
chr16	86,766,712	86,768,118	1407	6	LINC02188 *	Intergenic	1.59	1.1 × 10^−6^	0.0003
chr10	99,338,056	99,338,241	186	4	*ANKRD2*	Exon	1.18	2.1 × 10^−7^	0.0005
chr1	3,774,827	3,775,207	381	6	*DFFB*	Promoter	1.34	7.0 × 10^−7^	0.0008
chr6	39,760,607	39,761,596	990	6	*DAAM2*	Promoter	0.92	0.000002	0.0010
**DMRs detected with reduced DNA methylation levels in children sensitized to food allergens**									
chr10	128,810,484	128,810,905	422	3	*DOCK1*	Exon	−2.54	1.2 × 10^−6^	0.0013
chr17	72,462,417	72,463,081	665	6	*CD300A*	Promoter	−2.28	5.8 × 10^−6^	0.0038
chr17	75,315,486	75,315,668	183	6	*SEPT9*	Promoter	−1.70	1.2 × 10^−6^	0.0028
chr15	45,670,865	45,671,196	332	10	*GATM*	Promoter	−1.69	1.2 × 10^−5^	0.016
chr11	1,463,541	1,463,663	123	4	*BRSK2*	Promoter	−1.58	1.0 × 10^−5^	0.038
chr17	7,591,564	7,591,948	385	9	*WRAP53*	Promoter	−1.27	1.4 × 10^−5^	0.016
chr19	49,223,814	49,224,166	353	5	*RASIP1*	3′ UTR	−1.13	4.1 × 10^−5^	0.049
chr3	149,469,835	149,470,420	586	7	*COMMD2*	Promoter	−1.12	3.6 × 10^−5^	0.026
chr7	157,809,235	157,809,597	363	5	*PTPRN2*	Intron	−1.01	3.6 × 10^−5^	0.042
chr2	179,387,853	179,388,065	213	3	*TTN-AS1* *MIR548N*	Promoter	−0.7	7.3 × 10^−7^	0.0015

* Gene with the closest transcription start site to the DMR. Chr = chromosome; bp = base pair.

**Table 5:** A summary of genes containing DMPs and/or DMRs associated with IgE sensitization to airborne and food allergens in ALADDIN.

Gene Symbol	Gene Name	Locus	Function	DMR for Airborne Sensitization	DMR for Food Sensitization
*RUFY1*	RUN and FYVE Domain Containing 1	5q35.3	Binds phospholipid vesicles containing phosphatidylinositol 3-phosphate and participates in early endosomal trafficking	M, CB, 2Y, 5Y	CB, 2Y
*DUSP22*	Dual specificity phosphatase 22	6p25.3	Activates the Janus kinase signaling pathway	5Y	2Y
*TUBGCP5*	Tubulin Gamma Complex Associated Protein 5	15q11.2	Microtubule binding	5Y	.
*CD300A*	CD300a Molecule	17q25.1	Negatively regulates TLR signaling via Myd88	2Y, 5Y	5Y
*AIF1*	Allograft Inflammatory Factor 1	6p21.33	May promote macrophage activation upon being induced by cytokines and interferon	5Y	.
*TTLL10*	Tubulin Tyrosine Ligase Like 10	1p36.33	Unknown	CB, 2Y	CB, 2Y, 5Y
*DOCK1*	Dedicator of cytokinesis 1	10q26.2	Guanine nucleotide exchange factor involved in cytoskeletal rearrangements and focal adhesion	5Y	5Y
*BRSK2*	BR Serine/Threonine Kinase 2	11p15.5	Mediates phosphorylation	2Y, 5Y	2Y, 5Y
*COMMD2*	COMM Domain Containing 2	3q25.1	May down-regulate the activation of NFκB	.	5Y
*RPTOR*	Regulatory associated protein of MTOR complex 1	17q25.3	Negatively regulates the mTOR kinase	M	.
*CCR9*	C-C Motif Chemokine Receptor 9	3p21.31	Chemokine receptor for CCL25. Functional specialization of immune responses in different segment of the gastrointestinal tract.	5Y	.
*SEPT9*	Septin 9	17q25.3	Cytokinesis? May play a role in the internalization of intracellular microbial pathogens	.	5Y
*IGF1R*	Insulin Like Growth Factor 1 Receptor	15q26.3	Activation of JAK/STAT signaling	CB	CB, 5Y

DMR = differentially methylated region. M = mother, CB = cord blood, 2Y = 2 years of age, 5Y = 5 years of age.

## Data Availability

Data is contained within the article.

## Supplementary Materials

Supplementary Materials can be found at https://www.mdpi.com/1422-0067/22/2/801/s1. Table S1: The specificity and fluorochrome labeling of the antibodies used in flow cytometry analysis of isolated PBMC, Table S2: The cell proportions in PBMC from the mothers and their children at 5 years of age according to sensitization or not to allergens as analyzed by flow cytometry, Table S3: The cell proportions in PBMC from mothers, cord blood and children at 2 years and 5 years of age according to lifestyle as analyzed by flow cytometry, Table S4: Characteristics of the BAMSE population with DNA methylation data, Figure S1: Following quality control analyses, a total of 18 samples were removed from the original dataset: 11 because of low intensity signal, 5 as their raw hyper-methylation values was strongly deviated toward low intensity ratios, and 2 for contamination of DNA material. (A) Reports control intensity probes distribution among the PBMC samples. Dots color is associated with array ID. Green and brown circles highlight 11 excluded samples. (B) Reports beta values distribution of all samples colored by age. The blue arrow points to five removed samples. (C) A multi-dimensional scaling (MDS) plot of the samples colored by sex (females reported in green and males in orange). The misplacing of the two samples CB-123 and B24-272 can be reconducted to a contamination of maternal DNA material, Spreadsheet File S1: DMPs associated with airborne and food sensitization in ALADDIN, Spreadsheet File S2: CpGs within DMRs associated with airborne sensitization in ALADDIN, Spreadsheet File S3: CpGs within DMRs associated with food sensitization in ALADDIN, Spreadsheet File S4: DMRs associated with airborne and food sensitization in ALADDIN, Spreadsheet File S5: DMRs detected in maternal PBMC samples associated with airborne sensitization at 5Y, Spreadsheet File S6: CpGs replicated in BAMSE.
