# Supplementary material for: DNA Methylation Levels in Mononuclear Leukocytes from the Mother and Her Child Are Associated with IgE Sensitization to Allergens in Early Life
Source: Int J Mol Sci. 2021 Jan 14;22(2):801. doi: 10.3390/ijms22020801 (PMC7830007; doi:10.3390/ijms22020801)
Supplement: Supplementary file 1 [file ijms-22-00801-s001.zip › Supplementary materials/Figure S1[2].docx]

**Figure S1**. Following quality control analyses, a total of 18 samples were removed from the original dataset: 11 because of low intensity signal, 5 as their raw hyper-methylation values was strongly deviated toward low intensity ratios, and 2 for contamination of DNA material. A) Reports control intensity probes distribution among the PBMC samples. Dots color is associated with array id. green and brown circles highlight 11 excluded samples. B) Reports beta values distribution of all samples colored by age. The blue arrow points to 5 removed samples. C) A multi-dimensional scaling (MDS) plot of the samples colored by sex (females reported in green and males in orange). The misplacing of the two samples CB-123 and B24-272 can be reconducted to a contamination of maternal DNA material.
