# Supplementary material for: DNA Methylation Levels in Mononuclear Leukocytes from the Mother and Her Child Are Associated with IgE Sensitization to Allergens in Early Life
Source: Int J Mol Sci. 2021 Jan 14;22(2):801. doi: 10.3390/ijms22020801 (PMC7830007; doi:10.3390/ijms22020801)
Supplement: Supplementary file 1 [file ijms-22-00801-s001.zip › Supplementary materials/Table S1-S4[2] 201027 AS.docx]

| Table S1. The specificity and fluorochrome labeling of the antibodies used in flow cytometry analysis of isolated PBMC. | | | |
| --- | --- | --- | --- |
| CD marker | **Fluorochrome** | **Clone/Isotype** | **Cell phenotype** |
| CD45 | VioBlue^®^ | 5B1/ mouse IgG_2a_ | Gating for all leukocytes in the PBMC sample and expressed as % of CD45^+^ cells |
| CD3 | APC | BW264/56/ mouse IgG_2a_ | CD3 was used for gating cell populations in combination with other markers |
| CD4 | PerCP | VIT4/ mouse IgG_2a_ | Gated as CD3^+^CD4^+^ and expressed as % of T helper cells |
| CD8 | APC-Vio 770 | BW135/80/ mouse IgG_2a_ | Gated as CD3^+^CD8^+^ and expressed as % of T cytotoxic cells |
| CD14 | FITC | Tük4/ mouse IgG_2a_ | Gated as CD3^-^CD14^+^ and expressed as % of monocytes |
| CD16 | PE | REA423/ human IgG_1_ | Gated as SSC/CD16^+^ and expressed as % of neutrophils |
| CD19 | PE-Vio770 | LT19/ mouse IgG1 | Gated as CD3^-^CD19^+^ and expressed as % of B cells |
| APC: Allophycocyanin; FITC: Fluorescein-5-isothiocyanate; PE: R-phycoerythrin; PerCP: Peridinin Chlorophyll Protein Complex; SSC: side scatter. | | | |

| **Table S2. The cell proportions in PBMC from the mothers and their children at 5 years of age according to sensitization or not to allergens as analyzed by flow cytometry.** | | | | | | | | | | |
| --- | --- | --- | --- | --- | --- | --- | --- | --- | --- | --- |
| **Cell-population** | | **IgE Sensitized^†^**  **% (n)** | | | **Non-sensitized**  **% (n)** | | | **P-value^‡^** | | |
| **CD3^+^ CD4^+^ T cells**  Mothers  Aeroallergens | | | 31.59 ± 2.23  (n = 20) | | 33.66 ± 1.52  (n = 50) | | | | 0.45 | |
| Children 5 years  Aeroallergens  Food allergens | | | 36.03 ± 1.45  (n = 35)  37.04 ± 2.24  (n = 17) | | 39.02 ± 1.37  (n = 36)  37.77 ± 1.13  (n = 54) | | | | 0.15  0.75 | |
| **CD3^+^ CD8^+^ T cells**  Mothers  Aeroallergens | | | 17.97 ± 1.39  (n = 20) | | 17.8 ± 0.99  (n = 50) | | | | 0.92 | |
| Children 5 years  Aeroallergens  Food allergens | | 19.78 ± 0.94  (n = 35)  17.83 ± 1.26  (n = 17) | | | 20.73 ± 0.78  (n = 36)  21.03 ± 0.67  (n = 54) | | | 0.43  0.17 | | |
| **CD3^-^CD19^+^ B cells**  Mothers  Aeroallergens | | 8.22 ± 0.55  (n = 20) | | | 7.76 ± 0.54  (n = 50) | | | 0.62 | | |
| Children 5 years  Aeroallergens  Food allergens | | 10.48 ± 0.97  (n = 35)  11.19 ± 1.41  (n = 17) | | | 9.85 ± 0.64  (n = 36)  9.84 ± 0.61  (n = 54) | | | 0.58  0.32 | | |
| **CD3^-^CD14^+^ monocytes**  Mothers | | 18.89 ± 2.18  (n = 20) | | | 19.1 ± 1.42  (n = 50) | | | 0.93 | | |
| Children 5 years  Aeroallergens  Food allergens | | 8.9 ± 0.68  (n = 35)  8.05 ± 0.84  (n = 17) | | | 8.64 ± 0.85  (n = 36)  8.98 ± 0.66  (n = 54) | | | 0.83  0.47 | | |
| **SSC/CD16^+^ neutrophils**  Mothers | | 4.0 ± 1.27  (n = 20) | | | 4.21 ± 0.84  (n = 50) | | | 0.89 | | |
| Children 5 years  Aeroallergens  Food allergens | | 3.07 ± 1.16  (n = 35)  4.31 ± 2.27  (n = 17) | | | 1.31 ± 0.32  (n = 36)  1.57 ± 0.33  (n = 54) | | | 0.14  0.05 | | |
| †Classified as sensitized if the IgE level was ≥0.35 kU_A_/L (Thermo Fisher Scientific) to at least one out of nine aeroallergens analyzed using Phadiatop^TM^ and to at least one out of six food allergens analyzed using a food mix.  ‡P-values were calculated by T-test (unpaired).  The percentage of the different cell-types of total CD45^+^ cells are given as mean value ± SEM. SSC: side scatter | | | | | | | | | | |
| **Table S3.** **The cell proportions in PBMC from mothers, cord blood and children at 2 years and 5 years of age according to lifestyle as analyzed by flow cytometry.** | | | | | | | | | |  |
| **Cell-population** | **Anthroposophic**  **% (n)** | | | **Partly anthroposophic**  **% (n)** | | **Non-anthroposophic**  **% (n)** | **P-value†** | | |  |
| **CD3^+^ CD4^+^ T cells**  Mothers | 31.75 ± 2.01  (n = 15) | | | 35.21 ± 2.06  (n = 28) | | 31.6 ± 2.18  (n = 27) | 0.41 | | |  |
| Cord blood | 37.97 ± 2.44  (n = 15) | | | 36.98 ± 2.06  (n = 26) | | 39.2 ± 1.77  (n = 23) | 0.33 | | |  |
| Children 2 years | 40.66 ± 1.77  (n = 14) | | | 39.52 ± 1.55  (n = 25) | | 38.0 ± 2.53  (n = 25) | 0.83 | | |  |
| Children 5 years | 38.69 ± 2.09  (n = 16) | | | 36.45 ± 1.48  (n = 28) | | 38.14 ± 1.8  (n = 27) | 0.29 | | |  |
| **CD3^+^ CD8^+^ T cells**  Mothers | 16.75 ± 1.56  (n = 15) | | | 18.44 ± 1.21  (n = 28) | | 17.86 ± 1.46  (n = 27) | 0.44 | | |  |
| Cord blood | 12.98 ± 1.32  (n = 15) | | | 11.51 ± 0.93  (n = 26) | | 14.51 ± 0.84  (n = 23) | 0.44 | | |  |
| Children 2 years | 17.0 ± 1.44  (n = 14) | | | 17.77 ± 1.12  (n = 25) | | 17.99 ± 1.02  (n = 25) | 0.54 | | |  |
| Children 5 years | 20.14 ± 1.19  (n = 16) | | | 19.69 ± 0.94  (n = 28) | | 20.96 ± 1.08  (n = 27) | 0.39 | | |  |
| **CD3^-^ CD19^+^ B cells**  Mothers | 8.11 ± 1.04  (n = 15) | | | 7.74 ± 0.56  (n = 28) | | 7.94 ± 0.74  (n = 27) | 0.33 | | |  |
| Cord blood | 7.76 ± 0.78  (n = 15) | | | 7.96 ± 0.64  (n = 26) | | 7.22 ± 0.73  (n = 23) | 0.56 | | |  |
| Children 2 years | 15.9 ± 2.07  (n = 14) | | | 14.92 ± 1.05  (n = 25) | | 12.86 ± 1.31  (n = 25) | 0.41 | | |  |
| Children 5 years | 9.87 ± 1.28  (n = 16) | | | 10.15 ± 0.78  (n = 28) | | 10.36 ± 1.07  (n = 27) | 0.56 | | |  |
| **CD3^-^ CD14^+^ monocytes**  Mothers | 21.01 ± 2.11  (n = 15) | | | 18.29 ± 1.71  (n = 28) | | 19.37 ± 2.22  (n = 27) | 0.33 | | |  |
| Cord blood | 14.56 ± 1.88  (n = 15) | | | 11.92 ± 0.97  (n = 26) | | 10.84 ± 1.18  (n = 23) | 0.31 | | |  |
| Children 2 years | 7.86 ± 0.86  (n = 14) | | | 7.93 ± 0.96  (n = 25) | | 8.04 ± 0.85  (n = 25) | 0.59 | | |  |
| Children 5 years | 8.73 ± 1.27  (n = 16) | | | 9.41 ± 0.89  (n = 28) | | 8.11 ± 0.82  (n = 27) | 0.32 | | |  |
| **SSC/CD16^+^ neutrophils**  Mothers | 5.52 ± 2.25  (n = 15) | | | 2.34 ± 0.41  (n = 28) | | 5.28 ± 1.21  (n = 27) | 0.54 | | |  |
| Cord blood | 6.63 ± 1.58  (n = 15) | | | 8.68 ± 2.14  (n = 26) | | 7.11 ± 1.86  (n = 23) | 0.63 | | |  |
| Children 2 years | 0.82 ± 0.24  (n = 14) | | | 0.85 ± 0.13  (n = 25) | | 3.08 ± 1.51  (n = 25) | 0.79 | | |  |
| Children 5 years | 1.38 ± 0.45  (n = 16) | | | 2.19 ± 0.54  (n = 28) | | 2.65 ± 1.47  (n = 27) | 0.39 | | |  |
| **†**The comparison of the percentage of each cell-populations in the three different lifestyles (anthroposophic, partly-anthroposophic and non-anthroposophic) was done with the with Kruskal-Wallis test.  The percentage of the different cell-types of total CD45^+^ cells are given as mean value ± SEM. SSC: side scatter. | | | | | | | | | |  |

| **Table S4. Characteristics of the BAMSE population with DNA methylation data.** | |
| --- | --- |
| **Total number of children analyzed with DNA methylation data (n=256)** | **Number (n)** |
| Age years, mean (SD) | 4.3 (0.195) |
| Sex, Male/Female | 139/117 |
| Airborne allergen sensitized by Phadiatop^†^ test, Yes/No | 56/200 |
| Food allergen sensitized by food mix (fx5)^†^ test, Yes/No | 58/198 |
| Positive Phadiatop and/or food mix (fx5), Yes/No | 84/172 |
| †Classified as sensitized if the IgE level was ≥0.35 kU_A_/L (Thermo Fisher Scientific).  Phadiatop includes a mixture of cat, dog, horse, birch, timothy, mugworth, *Dermatophagoides pteronyssinus* and *Cladosporium* allergens.  Food mix includes a mixture of milk, egg white, soya bean, peanut, fish and wheat allergens. | |
